# Translation and cross-cultural adaptation of a Nepali version of the Dutch Participation and Activity Inventory for Children and Youth (PAI - CY) with visual impairment

**DOI:** 10.1186/s41687-021-00342-w

**Published:** 2021-08-25

**Authors:** Srijana Adhikari, Ellen Bernadette Maria Elsman, Ruth Marie Antoinette van Nispen, Fleur van Rens, Radhika Upreti Oli, Suman S. Thapa, Gerardus Hermanus Maria Bartholomeus van Rens

**Affiliations:** 1 Tilganga Institute of Ophthalmology, PO Box 561, Gaushala, Kathmandu, Nepal; 2 Department of Ophthalmology, VU University Medical Centre and the Amsterdam Public Health Research Institute, PO Box 7057, 1007 MB Amsterdam, The Netherlands; 3 Murdoch University, Perth, Australia

**Keywords:** Nepal, Children, Visual impairment, Blindness, Participation and activity

## Abstract

**Background:**

Visual impairment is an important cause of disability in children. There is a lack of information on rehabilitation needs and low vision support services for children with visual impairment in Nepal. This is a pilot study designed to translate, culturally adapt and pre-test the Dutch version of the Participation and Activity Inventory for Children and Youth (PAI-CY) with visual impairment aged 7–17 years to develop a Nepali version.

Questionnaires (PAI-CY versions for 7–12 and 13–17 years) were translated using standardized methods and were culturally adapted by a panel of experts. They were pretested to evaluate comprehensibility and relevance among six children with visual impairment and blindness. Finally, participants completed a questionnaire evaluation form.

**Results:**

The translation and cultural adaptation process resulted in the adaptation of nine items to make them suitable for Nepali culture. Most children had comprehensibility problems with some specific items because of vocabulary, sentence structure and the composition of items. Most of the children were satisfied with the questionnaires.

**Conclusion:**

The study resulted in the development of a Nepali version of the PAI-CY. We worked with a small group of content experts and a small but representative sample of children which allowed us to use rigorous translation procedures to address language and cultural differences. A population based study has been planned to investigate the psychometric properties of these questionnaires.

## Background

Childhood blindness is an important cause of disability in children [1]. It has been listed as one of the priority diseases by the World Health Organization (WHO) [2]. The prevalence and etiology of childhood blindness differs between countries and regions [3–6]. It has been estimated that of the 1.4 million blind children, two thirds live in low income countries in Asia and Africa [7]. Children in low income countries mostly suffer from diseases which are either preventable or treatable. During the past few decades, causes of childhood blindness in developing countries have slowly shifted from malnutrition and infectious diseases such as vitamin A deficiency and congenital rubella syndrome to uncorrected refractive errors, congenital cataracts, retinopathy of prematurity and cerebral visual impairment. In high income countries, albinism, optic nerve diseases, cerebral visual impairment and hereditary retinal dystrophies dominate the list of causes of childhood blindness [8–10]. Although there is no national registry, there is a handful of data available on visual impairment and blindness among children in Nepal. An epidemiological study conducted in three ecological regions in Nepal has shown that the prevalence of childhood blindness and visual impairment among children 0 to 14 years of age is 0.067% and 0.10%, respectively [11]. Moreover it was shown that about 80% of children suffer from avoidable blindness with uncorrected refractive error being the leading cause. Between 2008 and 2011, a nationwide survey among 778 children from 67 integrated schools for the blind was performed in different parts of Nepal. Corneal diseases due to vitamin A deficiency followed by retinal and lens disorders were the most common causes of blindness and visual impairment in these children [12]. Finally, a disability registry in Nepal has shown that there are around 3000 children who attend special schools for children with visual impairment [13]. According to the United Nations Convention on the Rights of People with Disabilities, disabled individuals, irrespective of the extent of their disabilities have the right to participate fully within society and in community life [14]. Children are in their formative stage approximately until the age of 18 years. Limitations caused by a visual disability, but also the lack of proper low vision services and guidance might prevent children from participating in age appropriate activities. This may ultimately have a negative impact on their development and psychosocial wellbeing [15–18]. For example Kef S found that of adolescents with visual impairment had fewer friends and smaller personal networks compared to their sighted counterparts. These factors are known to be an important component of functional social development. These visually impaired adolescents had fewer friends than their sighted counterparts. However, Huurrte T et al. found that the psychosocial developmental outcomes of many adolescents with visual impairment was similar to the psychosocial development of their sighted peers. Yet some adolescents with visual impairment, girls in particular, required more support in their psychosocial development.

There are no designated low vision services or support services in Nepal for children with visual impairment. Until now no study has addressed the issue of the lack of these services in terms of children’s developmental needs, participation in activities or quality of life. Therefore, it is not clear if children with visual impairment in Nepal have specific needs which would enable them to participate more fully.

A well-designed measurement tool to identify participation needs in children with visual impairment is important for any country or community. Participation could be measured using patient-reported outcome measures (PROMs). Many PROMs for measuring participation and activity limitations in adults with visual impairment exist [19, 20], and some are available for children with disability in general [21]. The field of PROMs for pediatric populations with visual impairment is more novel. In recent years, several PROMs for children with visual impairment have been developed [22, 23], but they are not yet available in the Nepali language. Instead of developing a new PROM to measure the participation and activity limitations of children in Nepal, it is considered to be more efficient to translate and culturally adapt an existing PROM, which is known to possess good psychometric properties.

The Participation and Activity Inventory for Children and Youth (PAI-CY) is one of those PROMs, and was originally developed in a Dutch pediatric population [24–29]. The PAI-CY was designed to measure the participation needs of children with visual impairment. Important advantages of the PAI-CY are that it consists of various age versions, covering the entire age range from 0 to 18 years, and that from age 7 years and onwards self-report forms are available alongside proxy-report forms for caregivers. Its psychometric properties are extensively evaluated using modern measurement techniques.

Translation and adaptation of existing tools to a local language is practiced worldwide. However, a good translation approach should follow proper guidelines of translation, adaptation and pretesting procedures [30, 31]. The objective of this study is to translate the Dutch PAI-CY questionnaires for children aged 7–12 years and 13–17 years, to adapt items to make them relevant to the Nepali context, and pretest the questionnaires to determine its feasibility.

## Methods

The study was first approved by the local Institutional Review Committee which was then forwarded to and approved by the Nepal Health Research Council. The study adheres to the tenets of declaration of Helsinki. Written informed consent was provided by parents of participating children, whereas assent was provided by the children themselves.

### Instruments

The Dutch PAI-CY questionnaires were developed for four age groups which reflected the developmental age bands of the WHO; 0–2 years (proxy-report), 3–6 years (proxy-report), 7–12 (proxy and self-report) and 13–17 years (proxy and self-report) [25–28]. This study concerns the PAI-CY 7–12 and 13–17. The PAI-CY 7–12 consists of 54 items which cover nine domains of contextual meaning: play, social contact, mobility, leisure time, communication, school, self-reliance, acceptance/self-consciousness and finance. The PAI-CY 13–17 years consists of 55 items which cover eight domains, namely: leisure time, mobility, social contact, communication, school, self-reliance, acceptance, /self-consciousness and finance. The domains provide contextual meaning to respondents. The items have a four-point Likert-type scale: (1) not difficult; (2) slightly difficult; (3) very difficult and (4) impossible. Furthermore, if an item is not relevant for the child, the response not applicable can be selected, which is treated as a missing value. Each domain also entails one open ended section “explanation or comments” which gives children the freedom to report any other relevant issues.

### Translation

The translation process was carried out in two steps. First, questionnaires were translated from Dutch to English to create an English version. Second, the English version was translated to Nepali to create a final Nepali version.

**Fig. 1:**
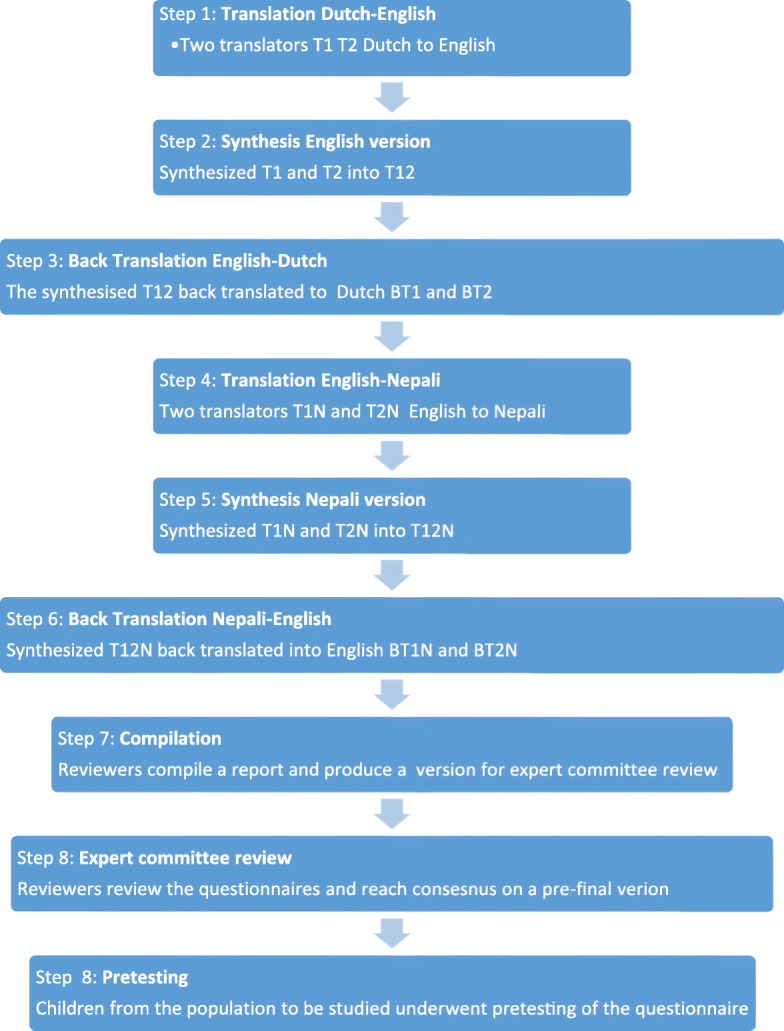
Translation and Pretesting

Figure 1 shows the translation process.

#### Translation from Dutch to English

##### Forward translation

Forward translation from Dutch to English was conducted by two translators. The first was a native Dutch speaker with advance mastery in English language. The translator had vast experience with measurement of PROMs and was involved in the development of the Dutch PAI-CY and related validation studies [23, 24]. The second is Dutch but residing in English speaking countries (UK, Australia) for many years and can be considered bilingual. She has been involved in the development of questionnaires and is an expert in the field of mobility and activity One English version was created by combining the two translations, along with a written report. This was finalized after review by the investigators of this study and reaching consensus by the two translators.

##### Backward translation

Backward translation from English to Dutch was conducted by two native Dutch speakers with advanced mastery of the English language. Both of them are university faculty members and are involved in various validation studies They were blinded to the original versions of the translated questionnaires. The back translations of both translators were then reviewed by the investigators and the two translators. The translated versions well matched the original Dutch questionnaires and no major discrepancies were found.

The study supervisor, who was fluent in both Dutch and English, and the translators convened on several occasions to develop a single English version for each questionnaire. This was done to eliminate any discrepancies in translation and to ensure expressions and concept equivalence. This generated the first reconciled English version of each questionnaire. The final English versions were then created after review and reaching consensus by the investigators and four translators. They were checked by a native English speaker from Australia.

#### Translation from English to Nepali

##### Forward translation

The final English version was forward translated into Nepali by two independent translators, both were ophthalmologists and had good mastery of English and Nepali. Both the translators were native Nepali speakers with advanced mastery of English language. They are involved in ophthalmology practice for the last 5 years. Both the translations were combined into final written report after review and reaching consensus by the investigators and the translators.

##### Backward translation

The backward translation was conducted by the two independent translators who were native Nepali speakers. One was an ophthalmologist working in the field for the last 5 years and the other was a public health expert with experience in eye research for the last 3 years. The backward translation did not show any major discrepancies from the original English versions of the questionnaires.

The final Nepali version were created after discussion between researchers, the public health experts and ophthalmologists. The Nepali version was further reviewed by the investigators to ensure that the questions had the intended meaning in the Nepali language.

### Cultural adaptation

The Dutch and Nepali researchers reviewed the final questionnaires together in a group discussion. It was checked whether items which represent Dutch life styles should be changed to items that better reflected the Nepali context. Four words in the items which represent Dutch life styles were changed to items that better reflected the Nepali context. The words “cycling”, “skateboard”, “kickscooter”,” preparing sandwitch” were changed to “walking”, “skipping”,“running” “Preparing bread and jam” respectively.

### Pretesting

After the translation and cultural adaptation process, a pretest of the Nepali version of the PAI-CY was conducted to check the comprehensibility and relevance of the questions. The pretesting was conducted using interview. The interviews were carried out by principal investigator of the study who is an ophthalmologist. Children with hearing impairment or cognitive abnormalities were excluded from the study Children with visual impairment and blindness according to WHO criteria were included in the study. Demographic information, visual acuity, and causes of blindness and visual impairment were collected using WHO prevention of blindness (PBL) form [32]. Blindness was defined as the best corrected visual acuity of less than 3/60 in the better seeing eye. Low vision (Moderate and severe visual impairment) was defined as the best corrected visual acuity of less than 6/18 but better than 3/60 in better seeing eye [33].

Item specific comprehensibility problems resulting from terminology, complex question structure, unclear formulation of questions, problems in understanding the response categories, and instructions were noted. Further pretesting helped to identify relevance of questions in Nepali context. Six children from the outpatient department of the Tilganga Institute of Ophthalmology, three with low vision and three with blindness, were invited to pretest the questionnaires. The questions were asked in a quiet room by the investigator. The responses were immediately filled out using a paper-and-pencil format. The time required to complete the interview for each child was recorded. Children were accompanied by either their mother or father. If children did not understand the questions, they could ask for assistance from their parents. The procedure was similar for both blind and visually impaired children.

Upon completion of questionnaires in pretest, children were asked to evaluate the questionnaires. There were five evaluation questionnaires regarding a, satisfaction b, length c, difficulty d, lacking questions e, relation to daily activities. The responses were recorded on the evaluation form, response categories included (a) yes (b) a little (c) no. were used in the evaluation form. During analysis these responses were categorized as either positive (+), neutral (±) or negative (−).

### Statistical analysis

Descriptive statistics were calculated for the demographic data (i.e. age, sex, visual acuity and the cause of blindness and low vision). The satisfaction regarding the questionnaires was assessed as were data in the evaluation form. The data were entered and analyzed in Microsoft Excel and data analysis was performed.

## Results

### Translation and cultural adaptation

During the course of translation, four items were changed from the Dutch version of the PAI-CY to adapt the questionnaires to the Nepali culture. In the Mobility domain, the item “Cycling to school” was changed to going to school by “walking” in both PAI-CY versions. The games using “skateboard” and “kick scooter” were changed to “skipping” and “running”. In the self- reliance domain “preparing a sandwich” was changed to “preparing bread and jam”, which is popular in Nepal and more easily available than “a sandwich”. From the group discussion with Dutch and Nepali researchers, the Dutch researchers who were the original developers of PAI-CY, mentioned that in the original validation study Dutch children stated missing various activities in the questionnaires [27, 28]. These items were discussed for their relevance to the Nepali context. Following this discussion,5 items were added in the PAI-CY 7–12 years (2 in “play”, 2 in “mobility” and 1 in “school” domain while 6 items were added to PAI-CY 13 to 17 years (1 in “mobility”, 1 in “school”,1 in “communication”,, 1 in ““self-reliance” and 2 in “acceptance/ self-consciousness” domain). Table 1 shows the changes made in different domain categories of the questionnaires.

**Table 1:** Results of changes made after translation and cultural adaptation

Age 7–12 years		
Domain	Question How difficult is it for you to:	Added or changed How difficult is it for you to:
**Play**		Play on the ground? (Added) Play indoor games like Ludo, block or doll games? (Added)
**Mobility**	Cycle independently (to school or a friend)? Participate in high speed games (e.g. kick biking, skating)?	Walk to school or a friend’s house? (Changed) Participate in high speed games (e.g. skipping, running)? (Changed) Cross road with traffic at night? (Added) Find your way in unknown environment? (Added)
**School**		Finish your homework independently? (Added)
**Self- reliance**	Make a sandwich?	Make a jam and bread? (Changed)
Age 13–17 years		
**Mobility**	Cycle independently (to school or a friend)?	Walk to school or a friend’s house? (Changed) Cross road with traffic at night? (Added)
**School**		Take part in outside school activities like inter-school sports competitions? (Added)
**Communication**		Recognize other people? (Added)
**Acceptance/self- consciousness**		Do your activities without getting fatigued? (Added) Divide your energy during the day? (Added)
**Self- reliance**		Pay attention to your facial care? (Added)

### Pretesting

The six children who were enrolled in the study were between 7 and 17 years of age. The age of children, visual acuity, anatomical site of the primary ocular diagnosis and mean duration to fill in the questionnaires are presented in Table 2.

**Table 2:** Characteristics of participants

	7–12 years (*n* = 3)	13–17 years (*n* = 3)
Mean Age in years	8.6	15.6
**Visual acuity**		
Blind	1	2
Low vision	2	1
**Ophthalmic diagnosis**		
Retina	2	1
Optic Nerve	1	
Lens		1
Cornea		1
Duration in minutes		
Mean (Range)	33.3 (25–45)	35.0 (20–45)

### Comprehensibility

None of the participants had any major issues answering the questions. However, all children reported some difficulties in the interpretation of words or phrases in some items. In the “acceptance/ self-reliance” domain children had difficulty in interpreting the phrase “to accept that you cannot do certain things/activities,” as “certain things” was deemed vague and non-specific. Hence it was advised to specify “certain things” like sports, or play music etc. This was applied to items in both age groups. In the age group 7–12 years, all three children required their parent’s help to answer questions when they were unsure about the answer. In the “Communication” domain, children needed their parents help in three items: “tell your parents what you want to say, while they understand what you mean”” talk about feelings (for example: if you feel scared or sad or happy,) do you tell that to your parents” and “notice how other children are feeling”. In the “Acceptance /Self-consciousness” domain children needed parents help with the item “empathize with others (for example: do you understand why a child in your class may sometimes be sad or upset)”.

One child suggested adding an open-ended question to the item related to music. In the age group 13–17 years all children had difficulties in interpreting the question on “handle or dealing with feelings of love” in the “social contact” domain. Therefore, this item was changed to “express the feeling of love”. A summary of the pretesting results is described in Table 3.

**Table 3:** Results of pretest in terms of comprehensibility

Subcategory	Description of problem	Domain/ Item no	Possible solution
Problem with instruction	Younger children had difficulty to answer questions and took mother’s help	Communication/ 1,3,9 **Item 1**“How difficult is if for you to tell your parents what you want to say; while they understand what you mean” **Item 3**:How difficult is it for you to talk about feelings (for example: if you feel scared or sad or happy,) do you tell that to your parents”; **Item 9**“How difficult is it for you to notice how other children are feeling Acceptance/ 4 How difficult is it for you to empathize with others (for example: do you understand why a child in your class may sometimes be sad or upset)”?	Younger children can answer questions with the help of caregiver
Item specific problem	The question was too vague. How difficult is it for you to accept that you can’t do “certain things”	Acceptance Self-reliance/ 3	“Certain things” to be specified like read, play etc. The question was modified to “How difficult it is for you to accept that you can’t do certain things, for example read, play etc.”
Item specific problem	How difficult for you to handle or deal with feeling of love	Social contact / 7	The question was modified to: How difficult it is it for you to “express feeling of love”

Based on the analysis of the evaluation form, it seemed that most children were satisfied with the questionnaires Table 4 describes the results of the evaluation form. However, the response was “neutral to negative” regarding the length of the questionnaires.

**Table 4:** Results of Evaluation of questionnaires by children and young adults (All questions)

How did you like this interview/ how satisfied are you with the interview	Rating	No of children	Only one child found the process tiring and expressed some dislike
	+	5	
	±	1	
	–	0	
Did you find this interview lengthy?	+	1	Most of the children found the questionnaire too long
	±	1	
	–	4	
Was it difficult to answer questions?	+	0	All children found the questions a little difficult
	±	6	
	–	0	
Any questions you want to add?	+	0	No one wanted to add any questions
	±	0	
	–	6	
How do the questions relate to your daily activities^a^	+	3	
	±		
	–		

^a^Asked to children aged 13–17 years

## Discussion

In our study, we translated two sets of PAI-CY questionnaires for two different age groups, 7–12 years and 13–17 years. The PAI-CY was translated from Dutch to English and from English to Nepali. A two–step translation process had to be followed because of a lack of experts who were able to translate directly from Dutch to Nepali and vice versa and were also knowledgeable with respect to the construct and population. Some cultural adaptation of items was required to enhance the applicability of the questionnaire within the Nepali context. For example, cycling is a very common form of transportation for children in the Dutch society. However, in Nepal it is sometimes used by children as recreational activity, but only few children use cycling as mode of transportation to go to school or other places. Also a few items which were not present in the Dutch PAI-CY were added to the Nepali version to better represent the live experiences of children with visual impairment in Nepal. These items were added based upon suggestions made by the Dutch children after extensive validation of questionnaires and after Dutch and Nepali researchers affirmed their applicability to the Nepali culture. Hence by common consensus and in collaboration with Dutch developers these items were added in the questionnaires. The Dutch developers recommended to include these items but not to include them in the scoring until validity and reliability is demonstrated. We are planning to study the reliability and validity of these tools including the added items in larger population of children.

Pretesting is a method of checking that the tool performs as intended and is understood by those individuals who are supposed to respond to it [34, 35], .Pretesting was performed in blind and visually impaired children to check the comprehensibility and reliability of questionnaires. During the process, there were minor issues encountered which have been changed accordingly. However, one major comprehensibility issue we encountered was that younger children were not able to answer items in some domains independently. They needed their parents help to answer those questions. Maybe these age groups in Nepal are less expressive or not confident enough to answer those questions. Therefore, it is important for children in this age group to have an adult present during completion of the questionnaires to ensure accurate responses.

The evaluation showed that most of the children were satisfied with the questions, which is similar to the pilot study conducted in Dutch children [23]. However, the length of the questionnaires was deemed an issue as most children indicated the administration took too long. This problem of endurance may be overcome by providing training to the personnel taking the interview and applying measures to keep children interested during the interview as well as providing a break if needed.

Our pilot study addresses a gap in the literature with regards to the lack of scientifically valid measurement tools to identify rehabilitation needs in blind and visually impaired children in Nepal. Although the LV Prasad Eye Institute in India has developed and validated a questionnaire which is probably more tailored towards the Nepali context, it measures the construct of functional vision, which is related to participation but does not assess children’s participation needs [36]. The development of measurement instruments or questionnaires is a research process that includes a number of carefully planned stages. Culturally adapting measurement tools from one cultural group to another can be a challenging task [37]. It should follow certain standard guidelines [38] (http://www.who.int/substance_abuse/research_tools/translation/en/). We were able to work with a small group of content experts and with a small but representative sample. This allowed us to use rigorous translation procedures to address language and cultural differences which is often overlooked in studies using translated questionnaires [39, 40]. A larger population-based study has been planned to further develop and validate the Nepalese PAY-CY questionnaires, by studying their psychometric properties as a quantitative instrument to assess the participation needs of Nepali children with visual impairment or blindness. Furthermore, this pilot study provided us with valuable experiences for selecting and recruiting participants and administering the PAI-CY questionnaires.

## Conclusion

This pilot study is an initial, but an important step in the validation of questionnaires in Nepalese children. Although there were some minor comprehensibility and response category problems, the questionnaires were considered to include items that reflect the most important activities and participation aspects of children with visual impairment. Most of the items and response categories were clear to the users. The adaptations made to the questionnaires after pretesting are likely to improve satisfaction with the content, the clarification of questions, and satisfaction with the questionnaires with regard to compiling a rehabilitation plan.

## Data Availability

The datasets used and/or analyzed during the current study are available from the corresponding author on reasonable request.

## Abbreviations

PAI-CY: Participatory and Activity Inventory Children and Youth
WHO: World Health Organization
PROM: Patient Reported Outcome Measure
